# Educational disparities in Brazil may interfere with the cognitive performance of Parkinson's disease patients

**DOI:** 10.1590/1980-5764-DN-2022-0084

**Published:** 2023-11-20

**Authors:** Danielle Pessoa Lima, Janine de Carvalho Bonfadini, Alexandre Henrique Silva Carneiro, Samuel Brito de Almeida, Antonio Brazil Viana, Ana Cecília Nogueira e Silva, Jarbas de Sá Roriz, Pedro Braga

**Affiliations:** 1 Universidade Federal do Ceará, Departamento de Clínica Médica, Divisão de Geriatria, Fortaleza CE, Brazil.; 2 Universidade Federal do Ceará, Departamento de Clínica Médica, Divisão de Neurologia, Fortaleza CE, Brazil.; 3 Universidade de Fortaleza, Faculdade de Medicina, Fortaleza CE, Brazil.; 4 Universidade Federal do Ceará, Complexo Hospitalar da Fortaleza, Unidade de Pesquisa Clínica, Fortaleza CE, Brazil.; 5 Universidade Estadual do Ceará, Faculdade de Medicina, Fortaleza CE, Brazil.

**Keywords:** Parkinson Disease, Cognitive Dysfunction, Mental Status and Dementia Tests, Doença de Parkinson, Disfunção Cognitiva, Testes de Estado Mental e Demência

## Abstract

The prevalence of cognitive impairment in Parkinson's disease (PD) is about 20% to 60%. The Mini-Mental Status Examination (MMSE) is the most used cognitive screening test.

**Objective:**

To evaluate the influence of clinical and demographic characteristics, specifically the education level, on the MMSE score in PD patients of a northeast Brazilian sample.

**Methods:**

We performed a cross-sectional study of 198 PD patients at a Movement Disorders outpatient clinic in Fortaleza, CE, Brazil. Participants were assessed by detailed clinical history, modified Hoehn and Yahr staging (HY), geriatric depression scale (GDS) and MMSE.

**Results:**

We found that 68% of patients had MMSE scores below the Brazilian thresholds, which were based in Brucki et al. study (2003). There was a statistically significant difference in the bivariate analysis between educational level and cut-off classification for MMSE. More years of formal schooling were associated with more patients scoring below threshold. We found that 75%, 68.8%, and 79.7% of individuals with more than 11, 9 to 11, and 4 to 8 years of formal schooling, respectively, were below the suggested Brazilian Brucki's threshold. GDS and age were negatively correlated with total MMSE and all its domains. There was no correlation between disease duration and MMSE. Subjects with hallucinations had lower scores.

**Conclusion:**

Most of the sample had lower performance according to Brazilian thresholds, but there was no control group and no neuropsychological test in this study. Further studies in northeast Brazil are needed to review MMSE cut-off values.

## RESUMO.

A prevalência de comprometimento cognitivo na doença de Parkinson (DP) é de cerca de 20 a 60%. O Miniexame do Estado Mental (MEEM) é o teste de rastreio cognitivo mais utilizado.

**Objetivo:**

Avaliar a influência de características clínicas e demográficas, especificamente a escolaridade, no escore do MEEM em pacientes com DP de uma amostra do nordeste brasileiro.

**Métodos:**

Realizamos um estudo transversal com 198 pacientes com DP em um ambulatório de Distúrbios do Movimento em Fortaleza. Os participantes foram avaliados por história clínica detalhada, estadiamento modificado de Hoehn e Yahr (HY), escala de depressão geriátrica (EDG) e MEEM.

**Resultados:**

Encontramos 68% dos pacientes com escores do MEEM abaixo dos limiares brasileiros baseados em estudo de Brucki et al. (2003). Houve diferença estatisticamente significativa na análise bivariada entre a escolaridade e a classificação de corte para o MEEM. Mais anos de escolaridade foram associados a mais pacientes com pontuação abaixo do limiar. Constatamos que 75, 68,8 e 79,7% dos indivíduos com mais de 11, nove a 11 e quatro a oito anos de escolaridade, respectivamente, estavam abaixo dos limiares sugeridos pelo estudo brasileiro de Brucki et al. (2003). A EDG e a idade correlacionaram-se negativamente com o MEEM total e todos os seus domínios. Não houve correlação entre a duração da doença e o MEEM. Indivíduos com alucinações tiveram pontuações mais baixas.

**Conclusão:**

A maioria da amostra apresentou desempenho inferior aos limiares, mas não houve grupo controle e nem teste neuropsicológico neste estudo. Mais estudos no nordeste do Brasil são necessários para revisar os valores de corte do MEEM.

## INTRODUCTION

Parkinson's disease (PD) is characterized by motor and non-motor symptoms (NMS) such as olfactory disturbance, autonomic dysfunction, cognitive impairment, mood and sleep disorders, fatigue, apathy, pain, and others^1^. NMS are very common in PD^2^, although they are not often investigated or declared during routine consultations^3^. Previous research has established that NMS have greater impact on quality of life than motor symptoms. These symptoms are directly associated with reduced patient quality of life and increased caregiver burden^4^.

Cognitive decline is a very prevalent NMS in PD and therefore an important therapeutic target. The estimated prevalence of mild cognitive impairment (MCI) is between 20 and 60% in diagnosed PD patients^5^. The prevalence of dementia increases with the progression of the disease, for which the cumulative incidence is 75 to 90%^6^.

Cognitive changes in PD are heterogeneous among patients. According to a previous task force, single domain MCI is more prevalent than multiple domain MCI, and non-amnestic single domain MCI is more common than amnestic single domain MCI in PD^7^. Cognitive impairment of the brain's prefrontal region is very common in PD, which include executive functioning skills (planning, working memory, mental flexibility, activation of remote memory, reinforcement learning, and inhibitory response)^8^. Memory recall is altered in PD patients due to compromised frontal attention function and cognitive flexibility^9^. Another deficit occurs in visual-spatial capacity due to posterior cortical involvement^5^.

Clinical monitoring of the cognitive functioning of PD patients is crucial for early detection of mild cognitive impairment because these patients tend to be at a higher risk for developing dementia^7^. Out of many currently available screening tests, the most known and used is the Mini-Mental Status Examination (MMSE)^10^. The performance of PD patients on the MMSE has varied considerably over time in longitudinal studies, indicating its relevance for tracking cognitive decline^11^. However, previous research determined that the MMSE demonstrated low sensitivity, according to the cut-off recommended by the International Parkinson Disease and Movement Disorder Society (26 points)^12^. Another issue to be considered regarding the MMSE is its educational bias, as specific items of the MMSE are influenced by education. Most of the Brazilian population has less than 8 years of formal schooling. Of note, Brazil's northeast population presents worse socioeconomic and educational conditions than those in the south and southeast^13^ parts of the country. The purpose of this study is to evaluate the influence of clinical and demographic characteristics, especially the education level, on the MMSE score in PD patients of a northeast Brazilian sample.

## METHODS

We performed a cross-sectional study of 198 PD patients at the Movement Disorders outpatient clinic in a public tertiary hospital in Fortaleza, located in the northeast region of Brazil. The inclusion criterion was diagnosis of PD according to the Movement Disorders Society^14^. The criteria for excluding the subjects were as follows: dementia of any etiology, sensory deficit (hearing or sight), or motor impairment that hinders cognitive assessment.

We consecutively drew the patients from the outpatient clinic's medical appointment scheduling list from March to December 2018. The patients were regularly monitored by the team of two neurologists and one geriatrician. Those who had the diagnosis of mild cognitive impairment or dementia described in the chart were excluded from the study. After the screening revision of the chart, the eligible patients were invited to participate in the study while they were waiting to be attended by the physician. The MMSE was applied in an exclusive office for this purpose by a psychologist trained and supervised by a neuropsychologist. A neurologist or geriatrician from our outpatient clinic diagnoses dementia based on the clinical assessment of the patient and information provided by the patient's family concerning their functional level. When there was doubt of mild cognitive impairment or dementia, they requested a neuropsychological evaluation.

After obtaining the written informed consent from the patients, we used an ethnographic interview to collect sociodemographic and clinical data, such as gender, age, disease duration, antiparkinsonian treatments such as L-dopa (L-dopa/carbidopa, L-dopa/benserazide and L-dopa controlled release formulations), COMT inhibitors (entacapone), MAO-B inhibitors (rasagiline), amantadine, and dopamine agonist (pramipexole). We adopted the standardized equivalent dose of levodopa (LED) formulae established by a previous systematic review^15^ to compare the dosage of different antiparkinsonian medications.

Our hypothesis was that the low education quality of our sample from northeast Brazil would comprise their cognitive performance. We separated the sample into two groups to examine the parameters related with their MMSE cognitive performance: those who scored above and below the Brucki cut-off values.

We evaluated the current history of depression using the Diagnostic and Statistical Manual of Mental Disorders (DSM-V). We also used the modified Hoehn and Yahr staging (HY) to assess the severity of PD, the geriatric depression scale of 15 questions (GDS-15) to assess depressive symptoms, and the MMSE to screen cognition.

The final MMSE score is the sum of correct answers in the following domains: temporal orientation (0 to 5 points), spatial orientation (0 to 5), registration (0 to 3), calculation (0 to 5), recall (0 to 3), language (0 to 8), as well as visuospatial function (0 to 1). The maximum score is 30 and the minimum score is zero. The lower the score, the more significant the impairment. We classified the final score based on the cut-offs related to years of formal schooling proposed by Brucki et al.^16^ The detailed classification goes as follows: 20 for illiterate subjects; 25 for 1 to 4 years of schooling, 26.5 for 5 to 8 years, 28 for 9 to 11 years, and 29 for more than 11 years. All patients were evaluated taking into account the stage of the disease (modified HY scale), depression symptoms, and cognition (MMSE) during “on” phases.

### Ethics

All patients gave their written consent to store and use clinical samples for research purposes. The study was approved by the local ethics committee (register number 91075318.1.0000.5045) and conducted according to ethical standards of the human experimentation committee and the guidelines of the Declaration of Helsinki.

### Statistical analysis

Data for numerical variables were presented as means, standard deviations, and medians. Data for categorical variables were described as frequencies and prevalence rates. A Mann-Whitney U-test analysis was used to compare two groups: those above and those below the Brucki's cut-off value, because independent variables were not normally distributed. We used the Pearson's chi-squared and Fisher's exact tests to investigate the association between categorical variables. Statistical analysis was performed using the JAMOVI statistical software program (Version 0.9).

## RESULTS

**Figure 1:**
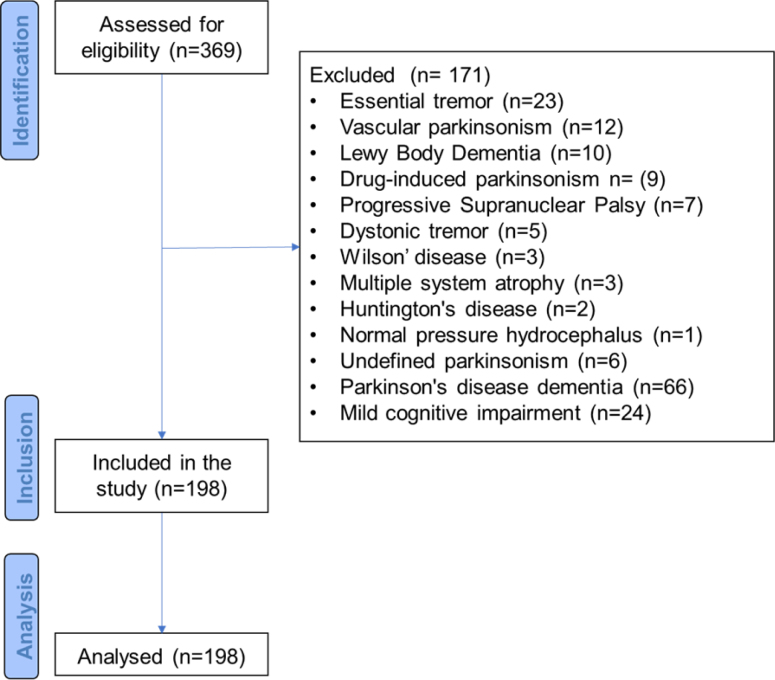
Study flowchart.

The study sample comprised 198 patients attending the outpatient clinic (Figure 1). A total of 109 (55%) patients in the sample were men, with a mean age of 65 years, and over half of them (128; 64.6%) were married. Also, 130 patients (65.6%) had eight or less years of formal education. Most of the patients (151; 76.2%) were classified as HY 2 to 3, and the mean disease duration was 8.9±6.6 years. Motor fluctuation and sleep complaints were the most common clinical features as reported by 103 (52%) and 109 (55%) of patients, respectively.

Most of the PD patients (135; 68%) had MMSE scores below the Brazilian threshold. Table 1 provides the bivariate analysis between these two categorical groups (below and above cutoff) and sociodemographic and clinical features. There was a statistically significant difference in bivariate analysis between educational level and cut-off classification (p=0.002), in which illiterate patients had a lower prevalence of people below the cut-off (35%), diverging from the other categories. In this respect, more years of formal schooling were associated with lower scores, with 75, 68.8, and 79.7% of individuals with more than 11, 9 to 11, and 4 to 8 years of formally schooling, respectively, being below the proposed Brazilian threshold.

**Table 1:** Comparison of sociodemographic and clinical characteristics between patients below and above Mini-Mental Status Examination cutoff score.

Variable		Total	Below cutoff (%)	Above cutoff (%)	p-value
Gender					
	Male	109	68 (62.4)	41 (37.6)	0.053*
	Female	89	67 (75.3)	22 (24.7)	
Age (years)					
	<60	41	29 (21.5)	12 (19)	0.7755
	≥60	157	106 (78.5)	51 (81)	
Marital status					
	Married	128	86 (67.2)	42 (32.8)	0.190†
	Single	34	21 (61.8)	13 (38.2)	
	Common-law marriage	4	2 (50)	2 (50)	
	Widow	18	15 (83.3)	3 (16.7)	
	Divorced	11	10 (90.9)	1 (9.1)	
Years of education					
	Illiterate	20	7 (35)	13 (65)	**0.002** *
	1–4	41	24 (58.5)	17 (41.5)	
	4–8	69	55 (79.7)	14 (20.3)	
	9–11	32	22 (68.8)	10 (31.3)	
	>11	36	27 (75)	9 (25)	
Disease duration (years)					
	<8	90	59 (65.6)	31 (34.4)	0.7269
	≥8	84	58 (69)	26 (31)	
Hoehn and Yahr stage					
	HY 0	1	1 (100)	0 (0)	0.253†
	HY 1	18	13 (72.2)	5 (27.8)	
	HY 1.5	6	2 (33.3)	4 (66.7)	
	HY 2	75	46 (61.3)	29 (38.7)	
	HY 2.5	32	22 (68.8)	10 (31.3)	
	HY 3	44	34 (77.3)	10 (22.7)	
	HY 4	18	14 (77.8)	4 (22.2)	
	HY 5	3	3 (100)	0 (0)	
Geriatric depression scale			6 (3–9)	5 (2.5–8)	0.522‡
Age			66.42±14.01	63±12.84	0.063‡
Hallucinations					
	Yes	19	15 (78.9)	4 (21.1)	0.249*
	No	152	100 (65.8)	52 (34.2)	
Motor fluctuations					
	Yes	103	69 (67)	34 (33)	0.932*
	No	71	48 (67.6)	23 (32.4)	
Depression					
	Yes	80	56 (70)	24 (30)	0.537*
	No	93	61 (65.6)	32 (34.4)	
Dyskinesia					
	Yes	59	43 (72.9)	16 (27.1)	0.256*
	No	115	74 (64.3)	41 (35.7)	
Sleep complaints					
	Yes	109	72 (66.1)	37 (33.9)	0.666*
	No	65	45 (69.2)	20 (30.8)	
Levodopa equivalent dose			1100 (600–2000)	1000 (600–1700)	0.929

Notes: Data expressed as percentages; means±standard deviation for normally distributed data and medians (25th–75th) for non-normally distributed data; in bold p<0.05.

* Pearson's chi-squared test;

† Fisher's Exact test;

‡ Mann-Whitney test.

The MMSE total score and domains were analyzed for correlation with GDS, age and disease duration, as shown in Table 2. GDS was negatively correlated with total MMSE score (r=-0.233; p=0.002), and with the following domains: temporal orientation (r=-0.207; p=0.006), spatial orientation (r=-0.192; p=0.011), and calculation (r=-0.214; p=0.005). Age was negatively correlated with total MMSE score (r=-0.329; p<0.001), and with the following domains: temporal orientation (r=-0.197; p=0.009), spatial orientation (r=-0.169; p=0.026), registration (r=-0.173; p=0.022), calculation (r=-0.190; p=0.012), recall (r=-0.168; p=0.026), language (r=-0.221; p=0.003), and visuospatial function (r=-0.290; p<0.001). We evaluated the correlation of disease duration and MMSE total score considering only patients ≥65 years old, but there was no correlation (Table 3).

**Table 2:** Correlation analysis of Mini-Mental Status Examination with geriatric depression scale, age and disease duration.

Variable	GDS	Age	Disease duration
	rho (p-value)	rho (p-value)	rho (p-value)
Total score	**-0.233 (0.002)**	**-0.329 (<0.001)**	-0.043 (0.570)
Orientation to time	**-0.207 (0.006)**	**-0.197 (0.009)**	-0.124 (0.103)
Orientation to place	**-0.192 (0.011)**	**-0.169 (0.026)**	0.040 (0.599)
Registration	-0.008 (0.922)	**-0.173 (0.022)**	0.058 (0.442)
Attention and calculation	**-0.214 (0.005)**	**-0.190 (0.012)**	-0.017 (0.818)
Spontaneous recall	-0.093 (0.223)	**-0.168 (0.026)**	0.029 (0.705)
Language	-0.140 (0.066)	**-0.221 (0.003)**	-0.042 (0.578)
Visual construction	-0.062 (0.417)	**-0.290 (<0.001)**	-0.129 (0.087)

Abbreviations: GDS, geriatric depression scale. Notes: Data expressed as Spearman correlation coefficient (p-value); in bold p<0.05.

**Table 3:** Analysis for correlation of Mini-Mental Status Examination with disease duration in patients 65 years or older.

	Disease durationrho (p-value)	p
MMSE total	-0.062	0.539

Abbreviations: MMSE, Mini-Mental Status Examination. Note: Data expressed as Spearman correlation coefficient (p-value).

The analysis for correlation of absolute MMSE total score and its domains with sociodemographic and clinical features was also performed (Table 4). Male gender was associated with the higher score in the calculation domain (p=0.001); patients with higher years of education tended to have better results in temporal orientation, spatial orientation, calculation, language, and visuospatial function, as well as in total score (p<0.001); patients with depression had lower scores in spatial orientation (p=0.02), registration (p<0.001), calculation (p=0.05) and visuospatial function (p=0.004). Patients with hallucinations had a lower mean total score (p=0.005).

**Table 4:** Association analysis of Mini-Mental Status Examination total score and domains with sociodemographic and clinical features.

		Temporal orientation 0–5	Spatial orientation 0–5	Registration 0–3	Calculation 0–5	Recall 0–3	Language 0–8	Visuospatial function 0–1	Total
Gender									
	Male	5 (4–5)	5 (4–5)	3 (3–3)	3 (2–4)	2 (1–3)	7 (6–8)	0 (0–1)	25 (22–27)
	Female	5 (4–5)	5 (4–5)	3 (3–3)	2 (1–4)	2 (1–3)	7 (6–8)	0 (0–1)	24 (21–26)
	p-value	0.683	0.882	0.711	**0.001**	0.992	0.785	0.582	0.273
Years of education									
	Illiterate	4.5 (3.5–5)	4 (3.5–5)	3 (2.5–3)	1 (0.5–3)	2 (1–3)	5 (5–6)	0 (0–0)	20 (18–23.5)
	1–4	4 (4–5)	5 (4–5)	3 (3–3)	3 (1–3)	2 (2–3)	7 (6–8)	0 (0–1)	23 (20–26)
	4–8	5 (5–5)	5 (4–5)	3 (3–3)	2 (1–3)	2 (2–3)	7 (6–8)	0 (0–1)	24 (21–26)
	9–11	5 (4–5)	5 (5–5)	3 (3–3)	3.5 (2–5)	2 (1–3)	7.5 (7–8)	1 (0–1)	26 (24–28)
	>11	5 (5–5)	5 (5–5)	3 (3–3)	4 (3–5)	2 (1–3)	8 (7–8)	1 (0–1)	27(24–28.5)
	p-value	**<0.001**	**<0.001**	0.057	**<0.001**	0.865	**<0.001**	**<0.001**	**<0.001**
Depression									
	Yes	5 (4–5)	5 (4–5)	3 (3–3)	2 (1–4)	2 (1–3)	7 (6–8)	0 (0–1)	24 (21–27)
	No	5 (4–5)	5 (5–5)	3 (3–3)	3 (2–4)	2 (2–3)	7 (6–8)	0 (0–1)	25 (22–27)
	p-value	0.056	0.02	**<0.001**	0.05	0.085	0.662	**0.004**	0.136
Hallucinations									
	Yes	5 (3–5)	5 (4–5)	3 (2–3)	2 (0.5–3)	2 (1–2)	7 (6–8)	0 (0–0)	23 (18–25)
	No	5 (4–5)	5 (5–5)	3 (3–3)	3 (2–4)	2 (1–3)	7 (6–8)	0 (0–1)	25 (22–27)
	p-value	0.144	0.312	0.445	0.405	0.376	0.076	0.67	**0.005**

Note: Data expressed as median (25th–75th); in bold p<0.05.

Table 5 shows the analysis of MMSE versus years of schooling adjusted by HY and age. The patients with 4 to 8 years of schooling were 8.06 times more likely to have the MMSE below the cut-off than illiterate patients, while patients with more than 11 years of schooling were 6.29 times more likely to have the MMSE below the cut-off than illiterate patients.

**Table 5:** Association between years of schooling and Mini-Mental Status Examination adjusted by Hoehn and Yahr staging and age.

		OR (95%CI)	p-value
Years of education	1–4 years–Illiterate	2.667 (0.838–8.493)	0.097
	4–8 years–Illiterate	8.063 (2.56–25.401)	**<0.001**
	9–11 years–Illiterate	4.613 (1.331–15.987)	0.016
	>11 years–Illiterate	6.29 (1.812–21.841)	**0.004**

Abbreviations: OR, odd ratio; CI, confidence interval. Note: in bold p<0.05.

## DISCUSSION

Most of the PD patients in the current study scored below the Brazilian threshold on the MMSE. This finding was unexpected and suggests that the participants may have cognitive decline, or that Brucki's cut-offs based on education level may be inadequate for this sample. Previous studies have questioned the use of the MMSE for this population because it may not be sensitive enough to detect MCI and it may not effectively measure some of the cognitive areas most affected by PD^17^. Some patients in the prior studies had cognitive impairments that were not identified by the MMSE. However, these findings took place in high-income countries, a fact that distinguishes these studies from the one conducted in Brazil when income variables are considered. The MMSE may present limitations in low-income countries regarding the cognitive assessment of patients with PD^12^. Moreover, there are variances to consider even within the same nation.

Primary education in Brazil is very heterogeneous, with regional characteristics interfering in studies meant to evaluate cognitive performance^18^. People may reflect different learning skills despite having the same years of schooling due to social inequality^18^. This may explain why our study had a diverse response profile, especially in the higher education groups. These results raise the importance of more regional studies^19^. Higher education in Brazil is quite diverse, both in terms of public and private institutions. The National Assessment of Courses, an exam required for students graduating in most areas of studies, provides the greatest information on the quality of Brazilian higher education. The results of this test indicate wide quality differences, with the best results being found in the southeastern region^20^. The Brucki study was conducted in the southeastern region of Brazil, specifically in the state of Sao Paulo.

Another parameter to compare Brazilian regions is the municipal human development index (MHDI). This is a composite index that includes indicators for three aspects of human development: longevity, education, and income. The MHDI of 0.783 in Sao Paulo places it second in the Brazilian state rankings. Our study sample was taken from a health reference center in the State of Ceará, which has the lowest MHDI in the country. Ceará is ranked 17th in Brazil's State rankings, with a score of 0.682 due to historical and cultural factors^21^. New tests and screening approaches may need to be developed to match regional diversity^22^.

The results showed that the prevalence of PD patients below the cut-off score was lower among the illiterate sample of the population in the study when compared to those from the other educational levels. The illiterate group was the only one that was not majority below the MMSE threshold. This suggests that perhaps the problem of the score is more appropriate for illiterate subjects and less appropriate for those with higher education. It is important to note that there is great heterogeneity in the performance on cognitive tasks among illiterates in developing countries such as Brazil, probably due to different environmental demands, economic and socio-cultural backgrounds^18,22^.

A positive correlation between years of education with results in the MMSE total score and its domains was also observed, except for registration and spontaneous recall. These findings agree with the results of previous studies performed in other regions of Brazil^16^.

Older age is a risk factor for both MCI and PDD^5^. There was a negative correlation between age and MMSE total score and all its domains in our analysis. In contrast, there was no statistical significance between disease duration and MMSE total score, as well as for patients ≥65 years old. However, it is noteworthy that the number of patients with HY 4 and 5 was relatively small, probably due to the diagnosis of dementia and to the difficulty of getting to the clinic.

Studies about gender effect on MMSE scores are controversial^18^. We observed that male patients performed better in the cognitive domain of attention and calculation in our study. Nevertheless, it is complicated to determine whether this finding is due to biological aspects or due to a difference in education between men and women^23,24^.

Visual hallucinations are one of the most common neuropsychiatric symptoms in PD. A recently published review article^25^ illustrated that the presence of neuropsychiatric symptoms can affect cognitive skills in this patient population. Non-demented patients with hallucinations show faster rates of cognitive decline, and its presence in PD is considered a significant predictor of dementia^26^. Janzen et al. suggested that impairment of the pedunculopontine nucleus leading to cholinergic reduction causes visual hallucinations in PD^27^.

The individuals with hallucinations in our study had lower total MMSE scores. This correlation may reflect the cognitive impairment in PD, although other factors may also be related to the emergence of psychosis in PD, such as older age, longer duration of illness, higher severity of motor symptoms, and presence of sleep disturbances^28^.

Some limitations should be noted. There was no neuropsychological testing in the study, which is considered the gold standard for defining change in cognition^12^. Functional capacity would be another relevant piece of data, but we were unable to test it on every subject. Therefore, we could not determine whether the results suggest that education adjusted Brucki's cut-off scores are high for the sample of this study, or the participants have some degree of cognitive decline. Considering that PD is a very heterogeneous condition that has its stages, our study has the limitation of having evaluated the performance of the MMSE of the sample in different stages. We cannot conclude that the cause of the high prevalence of patients with performance below the cut-off is PD because the study did not have a control group.

In conclusion, according to our findings, 68% of our sample of Parkinson's disease patients had lower MMSE scores than those recommended by the 2003 Bucki study. It is impossible to determine whether this finding is the result of cognitive changes in PD or the inadequacy of the utilized cut-off values since there was no control group and no evidence of normal cognition through the neuropsychological test in this study. Further studies in northeast Brazil are needed to review MMSE cut-off values adjusted for years of formal schooling in PD patients.
